# Utility of quick sepsis-related organ failure assessment (qSOFA) to predict outcome in patients with pneumonia

**DOI:** 10.1371/journal.pone.0188913

**Published:** 2017-12-21

**Authors:** Martin Müller, Viviane Guignard, Joerg C. Schefold, Alexander B. Leichtle, Aristomenis K. Exadaktylos, Carmen A. Pfortmueller

**Affiliations:** 1 Department of Emergency Medicine, Inselspital, Bern University Hospital, University of Bern, Bern, Switzerland; 2 Department of Intensive Care Medicine, Inselspital, Bern University Hospital, University of Bern, Bern, Switzerland; 3 Department of Clinical Chemistry, Inselspital, Bern University Hospital, University of Bern, Bern Switzerland; National Yang-Ming University, TAIWAN

## Abstract

**Background:**

Despite on-going advances in medical treatment, the burden of disease of pneumonia remains high. We aimed to determine the association of the qSOFA score with in-hospital mortality, length of hospitalisation, and admission to the intensive care unit (ICU) in patients with pneumonia. Further, in a subgroup analysis, the outcomes were compared for qSOFA in comparison to other risk scores, including the CURB-65 and SIRS scores.

**Methods:**

In a retrospective analysis, admission data from the ED of the Bern University Hospital, Switzerland, were screened to identify patients admitted for pneumonia. In addition to clinical characteristics, qSOFA and CURB-65 scores and SIRS criteria were assessed and evaluated with respect to the defined study outcomes.

**Results:**

527 patients (median age 66 IQR 50–76) were included in this study. The overall in-hospital mortality was 13.3% (n = 70); 22.0% (n = 116) were transferred to the ICU. The median length of hospitalisation was 7 days (IQR 4–12). In comparison to qSOFA-negative patients, qSOFA-positive patients had increased odds ratios for in-hospital mortality (OR 2.6, 95%:1.4, 4.7, p<0.001) and ICU admission (3.5, 95% CI: 2.0. 5.8, p<0.001) and an increased length of stay (p<0.001). For ICU admission, the specificity of qSOPA-positivity (≥2) was 82.1% and sensitivity 43.0%. For in-hospital mortality, the specificity of qSOPA-positivity (≤2) was 88.9% and sensitivity 24.4%.

In the subgroup analysis (n = 366). The area under the receiver operating curve for ICU admission was higher for qSOFA than for the CURB-65 score (p = 0.013). The evaluated scores did not differ significantly in their prognostication of in-hospital mortality (p>0.05).

**Conclusions:**

The qSOFA score is associated with in-hospital mortality, ICU admission and length of hospitalisation in ED patients with pneumonia. Subgroup analysis revealed that qSOFA is superior to CURB-65 in respect to prognostication of ICU admission.

## Introduction

Pneumonia is defined as an acute infection of the pulmonary parenchyma, presenting with an acute infiltrate in the chest X-ray [1, 2]. Despite on-going advances in medical treatment, the burden of disease of pneumonia remains significant [3]. Even in developed countries, the incidence of pneumonia is still as high as 9.7 per 1000 persons, with a hospitalisation rate of 46.5% and 30-day mortality of 12.9% in patients with community-acquired pneumonia [3]. The case-fatality rate increases to over 50% in patients with pneumonia-related sepsis/septic shock [3, 4]. Therefore, early diagnosis of patients with pneumonia-associated sepsis/septic shock seems paramount.

In 2016, the third international consensus on sepsis definitions was published [5]. The aim of the consensus was to take into account emerging knowledge on immune function in sepsis, where sepsis was defined as a dysregulated host response to an external pathogen [5]. This new definition also aims to make criteria for diagnosis of sepsis more specific than the proposed SIRS criteria published in the prior 1992 2^nd^ international consensus, which are of rather low specificity and lead to a high percentage of false positives [5]. This led to a new sepsis definition; a change in sequential organ failure (SOFA) score of more than two points with either proven or suspected infection is diagnostic [5]. However, calculation of SOFA scores requires sequential laboratory work-up and is therefore not useful for bedside screening of patients with suspected sepsis [5, 6]. Therefore, the consensus committee has proposed the quick sequential organ failure score (qSOFA)—which is based on rapidly assessable vital parameters, including respiratory rate, mental status, and systolic blood pressure [6]. Since the proposal of qSOFA by the third international consensus, several studies have been performed to evaluate qSOFA in critically ill patients in various settings [6–17]. qSOFA is also useful in predicting the outcome prediction abilities in populations in the general emergency department (ED) or intensive care unit (ICU) [18]. However, it is also critical to validate this score in subgroups of critically ill patients and this has hardly been attempted. In addition, qSOFA must be compared to other disease-specific scores for outcome prediction.

The primary aim of this study is to evaluate the predictive performance of qSOFA in patients with pneumonia for the primary outcome of in-hospital mortality. Additional aims were to assess whether qSOFA-positive patients exhibited increased ICU admission rate and length of hospital stay. Moreover, we performed a subgroup analysis of these outcomes to compare the diagnostic power of qSOFA with that of CURB-65, SIRS and clinically diagnosed sepsis.

## Methods

### Setting

The study site was the emergency department (ED) of Bern University Hospital (Inselspital), with a caseload of more than 40,000 patients per year.

### Data collection and eligibility criteria

Our data analysis comprised adult patients admitted to the emergency department (ED) of Bern University Hospital between 1 June 2011 and 31 May 2013 and with the primary diagnosis of pneumonia. Patients were identified using the appropriate search string in the patients’ diagnosis or in the medical history field of our computerised patient database (E-Care, ED 2.1.3.0, Turnhout, Belgium).

All adult patients of 16 years or older presenting with the diagnosis of pneumonia were eligible for study inclusion. Patients with admissions not related to pneumonia, patients with duplicate records, or with incomplete data sets for the calculation of qSOFA were excluded from the study. Patients with restrictions to treatment, e.g. reasons against ICU admission, were not excluded from the analysis.

### Data extraction

The following clinical data were extracted from medical records: type of pneumonia (community-acquired/nosocomial), time since start of symptoms, history of fever, history of diarrhoea, history of delirium, history of myalgia, and risk factors for pneumonia [19] (chronic obstructive pulmonary disease (COPD), diabetes mellitus, liver disease, chronic renal failure, severe cardiac disease, immunosuppression, active neoplasia, smoking, alcoholism). In addition, we recorded whether patients were judged to be septic or diagnosed with sepsis by the attending emergency physician in the health reports. Furthermore, we recorded vital parameters (first recorded value), laboratory findings, microbiological sampling, and results, as well as duration of hospitalisation, intensive care unit (ICU) admissions, and in-hospital mortality. Demographic data, such as gender and age, were also assessed.

### Definitions

#### Pneumonia

Pneumonia was diagnosed by clinical examination together with laboratory and radiological work-up, as defined by our hospital standard of care. Appropriate microbiological evaluations and treatment were also based on this standard.

#### ICU admission

ICU admissions included primary ICU admissions (from the ED to the ICU), as well as secondary ICU admissions (from the general ward to the ICU).

#### qSOFA

The definition of the Surviving Sepsis Campaign 2016 was used to calculate the qSOFA score [5]: the qSOFA score was the sum of 1 point for a Glasgow Coma Scale (GCS) of 14 or less, 1 point for a systolic blood pressure of 100 mmHg or less, and 1 point for a respiration rate of 22/min or more.

#### CURB-65

The CURB score is a pneumonia-specific disease severity score that is widely used [20]. The CURB score stratifies patients into five strata, with increasing risk of mortality between the strata [20]. The CURB and its revised version, the CURB-65 score, have been validated in several studies and exhibit high negative predictive value in patients with pneumonia [20–22].

In the calculation of CURB-65, one point is scored for each of the following items: i) presence of confusion—defined as a GCS of 14 or less, ii) blood urea nitrogen of greater than 7mmol/l, iii) respiratory rate of 30/min or more, iv) a systolic blood pressure of less than 90mmHg or diastolic of 60mmHg or less, and v) age of 65 years or more [20]. A CURB-65 score of two or more differentiates patients with a high risk of mortality (9.1%) from those with a low (1.7%) risk of mortality. A CURB-65 score of 2 and more was therefore used as a cut-off and defined as positive.

#### SIRS

The systemic inflammatory response syndrome was part of the 1992 second international consensus on sepsis and is still widely used in clinical practise [23].

Criteria for diagnosis of SIRS are as follows: i) body temperature of above 38°C or below 36°C, ii) a heart rate of more than 90/min, iii) presence of hyperventilation defined by a respiratory rate of above 20/min or a partial pressure of CO_2_ of less than 32mmHg, iv) and a white blood cell count of above 12,000 cells/μL or less than 4,000/μl [23, 24].

#### Clinical judgement

If the diagnosis or medical history field of the patient’s records contained the terms”sepsis” or “septic”, one point was attributed for clinical judgement. This “clinical judgement” point was attributed solely on the basis of the doctor’s documentation, independent of whether the old or the new sepsis definition was fulfilled.

#### Threshold values

qSOFA, CURB-65, and SIRS criteria were considered positive when the patient scored two or more points [5, 21, 24].

### Ethical considerations

The study was approved by the regional ethics committee of the Canton of Bern, Switzerland (KEK: 09-07-13). Individual informed consent was waived by the ethics committee.

### Statistical analysis

Stata® 13.1 (StataCorp, The College Station, Texas, USA) was used for statistical analysis. All continuous variables were presented as medians with 25^th^- 75^th^ interquartile ranges (IQR). Categorical variables were shown with frequency accompanied by its proportion. The Mann-Whitney U test and the Kruskal-Wallis test were used to compare interval variables between two and more than two groups, e.g. the qSOFA-positive and qSOFA-negative groups. Comparisons of categorical variables between the qSOFA-positive and qSOFA-negative groups were performed with Fisher’s exact test. Predictive values of CURB-65 and qSOFA scores were calculated. The equality of the area under the receiver operating curves (AUC) was tested using the *roccomp* command to compare the diagnostic performance of the CURB-65 score, clinical judgement, and SIRS criteria with a qSOFA score in predicting in-hospital mortality or the need for ICU admission [25]. A p-value of <0.05 was considered significant.

## Results

### Patients’ demographics

**Fig 1:**
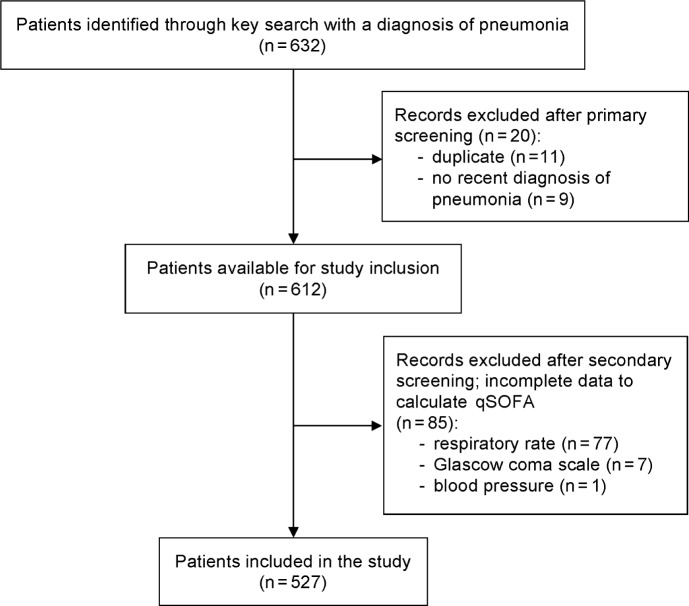
CONSORT flow chart.

Of the 612 patients with a diagnosis of pneumonia, 527 had complete datasets to calculate the qSOFA score and were eligible for study inclusion. The CONSORT flow chart is given (Fig 1).

The study population consists of 64.5% (n = 340) male patients; the median age was 66 years (IQR 50–76). Patient characteristics are given in Table 1. The diagnosis of community-acquired pneumonia was made in 476 patients (90.3%), while 51 patients (9.7%) suffered from nosocomial pneumonia. The most common risk factors were cardiovascular disease (n = 180, 34.2%) and smoking (n = 174, 33.0%), followed by COPD (n = 133, 25.2%). The median time since symptom onset was three days (IQR 2–5). Fever was the most common symptom (n = 289, 54.8%). Four hundred and sixty six (n = 466, 69.4%) patients were hospitalised. Overall in-hospital mortality was 13.3% (n = 80); 22.0% (n = 116) patients were admitted to the ICU and the median length of stay was 7 days (IQR 4–12).

**Table 1:** Patient characteristics.

Characteristics			
**N**		527	(100.0)
**Sex, [n (%)]**			
	Males	340	(64.5)
	Females	187	(35.5)
**Age (years), [median (IQR)]**		66	(50–76)
**Risk factors, [n (%)]**			
	Chronic obstructive pulmonary disease	133	(25.2)
	Diabetes mellitus	93	(17.6)
	Liver disease	38	(7.2)
	Chronic renal insufficiency	134	(25.4)
	Cardiovascular disease	180	(34.2)
	Neoplasia, any type	125	(23.7)
	Immunosuppressive therapy	101	(19.2)
	Smoking history	174	(33.0)
	History of alcoholism	39	(7.4)
**Time since symptom onset (days), [median (IQR)]**		3	(2–5)
**Clinical presentation, [n (%)]**			
	Fever	289	(54.8)
	Diarrhoea	45	(8.5)
	Delirium	38	(7.2)
	Myalgia	97	(18.4)
**Type of Pneumonia, [n (%)]**			
	Community acquired	476	(90.3)
	Nosocomial acquired	51	(9.7)
**Microbiological diagnostics, [n (%)]**			
	Blood culture obtained	416	(78.9)
	Blood culture *positive*	58	(13.9)
	Urine, legionella antigen positive*	11	(2.9)
	Urine, pneumococcus antigen *positive*^*#*^	35	(13.0)
	Sputum, obtained	110	(20.9)
	Sputum, *pathological*	89	(80.9)
**Clinical judgment, [n (%)]**			
	Clinically suspected sepsis	73	(13.9)
**Outcome parameter**			
	In-hospital mortality, [n (%)]	70	(13.3)
	ICU admission, [n (%)]	116	(22.0)
	Length of stay (days), [median (IQR)]	7	(4–12)

Urine antigen taken in *n = 271 / ^#^n = 192 patients

### qSOFA assessment

qSOFA score was positive in 86 patients (16.3%). Patients with a positive qSOFA score did not differ significantly in respect to age, sex, or risk factors for pneumonia from patients with a negative qSOFA score (all p>0.05). Characteristics of qSOFA positive and qSOFA negative patients are depicted in Table 2. Patients with a positive qSOFA had significantly fewer days since onset of symptoms (p = 0.016), a higher proportion of delirium and myalgia (p<0.001), and more often suffered from nosocomial pneumonia (p = 0.029) than patients who were qSOFA-negative. Furthermore, patients with a positive qSOFA score had significantly more positive blood cultures (p<0.001) and positive results of the pneumococcus antigen test (p = 0.003). There was a significant positive association between clinically suspected sepsis and a positive qSOFA score (p<0.001).

**Table 2:** Comparison of qSOFA-positive and qSOFA-negative patients.

Characteristics		qSOFA<2		qSOFA≥2		P
**n**		441	(83.7)	86	(16.3)	
**Sex, [n (%)]**						
	Male	292	(66.2)	48	(55.8)	
	Female	149	(33.8)	38	(44.2)	0.084
**Age, [median (IQR)]**		66	(52–75)	66	(50–80)	0.527
**Risk factors, all**						>0.05
**Days since symptom onset, [median (IQR)]**		3	(2–6)	2.5	(2–5)	0.016
**Clinical presentation, [n (%)]**						
	Fever	246	(55.8)	43	(50)	0.345
	Diarrhoea	34	(7.7)	11	(12.8)	0.139
	Delirium	20	(4.5)	18	(20.9)	<0.001
	Myalgia	92	(20.9)	5	(5.8)	<0.001
**Type of Pneumonia, [n (%)]**						
	Community acquired	404	(91.6)	72	(83.7)	
	Nosocomial acquired	37	(8.4)	14	(16.3)	0.029
**Diagnostics, [n (%)]**						
	Blood culture positive	37	(10.8)	21	(28.4)	<0.001
	Urine, legionella antigen	10	(3.2)	1	(1.6)	1.000
	Urine, pneumococcus antigen	22	(9.8)	13	(28.3)	0.003
	Sputum, pathological	75	(80.6)	14	(82.4)	1.000
**Clinical judgment, [n (%)]**						
	Clinically suspected sepsis	43	(9.8)	30	(34.9)	<0.001
**Length of stay (days), [median (IQR)]**		7	(4–12)	10	(6–16)	<0.001
**ICU admission, [n (%)]**		79	(17.9)	37	(43)	<0.001
**In-hospital mortality, [n (%)]**		49	(11.1)	21	(24.4)	<0.001

In comparison to qSOFA-negative patients, qSOFA-positive patients had increased odds for in-hospital mortality (OR 2.6, 95%:1.4, 4.7, p<0.001), ICU admission (3.5, 95% CI: 2.0. 5.8, p<0.001) and an increased length of hospital stay (7, IQR 4–12 vs. 10 IQR 6–16 days, p<0.001).

### Predictive value of qSOFA score

**Fig 2:**
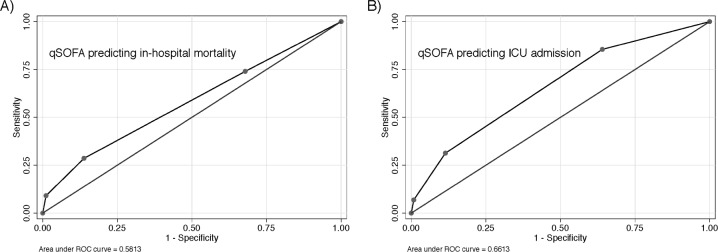
Receiver operating characteristic curve of qSOFA predicting A) in-hospital mortality, B) ICU admission.

The sensitivity of qSOFA in predicting in-hospital mortality and ICU admission was low—ranging from 15.5%-50.0% for in-hospital mortality and 28.9%-63.6% for ICU admission. The specificity ranged from 87.5%-89.5% for in-hospital mortality and 78.9%-90.1% for ICU admission (see Fig 2).

The area under the receiver operating curve (AUC) of qSOFA was 0.58 (95% CI: 0.52, 0.66) to predict in-hospital mortality and 0.66 (95% CI 0.62, 0.72) for ICU admissions. For a comprehensive comparison of the distribution of the outcomes in the different qSOFA categories, see Table 3.

**Table 3:** Length of hospital stay, ICU admission and in-hospital mortality in patients with different qSOFA score categories in patients with pneumonia (n = 527).

			Length of stay [days]			ICU admission			In-hospital mortality		
		*n*	Median (IQR)		p	Frequency (%)		p	Frequency (%)		p
**qSOFA**											
	0	191		6.0	(3–10)		19	(10.0)		20	(10.5)	
	1	250	8.0	(5–13)		60	(24.0)		29	(11.6)	
	2	75	10.0	(6–17)		30	(40.0)		15	(20.0)	
	3	11	6.0	(4–9)	<0.001	7	(63.6)	<0.001	6	(54.6)	0.001
**qSOFA**													
	<2	441	7.0	(4–12)		79	(17.9)		49	(11.1)	
	≥2	86	10.0	(6–16)	<0.001	37	(43.0)	<0.001	21	(24.4)	0.003
**Total**		527	7.0	(4–12)		22	(22.0)		70	(13.3)	

Patients with a qSOFA score of three points had a high ICU admission rate of 63.6%, with mortality of 54.6%. A qSOFA score of three points increases the probability of ICU admission by about 20% (LR+ 3.0) and of in-hospital mortality by about 28% (LR+ 4.4) [26].

The highest Youden index (= sensitivity + specificity-1) was found for a qSOFA score ≥3 in both in-hospital mortality (Youden index = 0.42) and ICU admission (Youden index = 0.43).

### Subgroup analysis—Comparison of qSOFA to other outcome prediction scores

A subgroup analysis was performed; this excluded patients with an incomplete dataset to calculate CURB-65 (blood nitrogen missing in 161 patients) or SIRS criteria (leucocytes missing in 2 and temperature in 18 patients).

Three hundred and forty-six patients (n = 346) with a median age of 68 years (IQR 58–77), an in-hospital mortality of 14.5% (n = 50), an ICU admission rate of 27.2% (n = 94), and a median length of stay of eight days (IQR 5–13) were included in the subgroup analysis.

qSOFA- and CURB-65-positive patients had increased in-hospital mortality (OR 2.7, 95% CI 1.3, 5.5, p = 0.002 and OR 3.3, 95% 1.8, 5.9, p<0.001), while clinical judgement and positive SIRS criteria were not associated with in-hospital mortality (OR 1.5, 95% CI 0.6, 3.1, p = 0.279 and OR 0.89, 95% CI 0.5, 1.8, p = 0.746).

For all evaluated 4 scores, score positive patients had a significant higher ICU admission rate and a significantly increased length of hospitalisation (p all <0.05).

Table 4 summarises the diagnostic performance of qSOFA, SIRS, and CURB-65 scores as well as clinical judgement in respect to in-hospital mortality and ICU admission.

**Table 4:** Diagnostic performance of different scores predicting A) ICU admission and B) In-hospital mortality (n = 346).

		Sensitivity [%]	Specificity [%]	Likelihood ratio +	Likelihood ratio -	Area under ROC
**A) ICU ADMISSION**						
	qSOFA*					
	≥1	34.2 (28.9)	87.5 (90.1)	2.7 (2.9)	0.8 (0.8)	
	≥2	48.4 (43.0)	77.7 (82.1)	2.2 (2.4)	0.7 (0.7)	
	≥3	70.0 (63.6)	74.1 (78.9)	2.7 (3.0)	0.4 (0.5)	0.668 (0.672)
	Suspected sepsis*					
	yes	54.7 (32.8)	79.1 (91.5)	2.6 (3.9)	0.6 (0.7)	0.629 (0.621)
	**CURB-65**					
	≥1	28.3	77.8	1.3	0.9	
	≥2	32.4	78.7	1.5	0.9	
	≥3	38.1	75.3	1.5	0.8	
	≥4	57.1	74.1	2.2	0.6	
	≥5	80.0	73.6	3.0	0.3	0.586
	**SIRS**					
	≥1	28.4	84.8	1.9	0.8	
	≥2	31.4	82.7	1.8	0.8	
	≥3	36.6	78.8	1.7	0.8	
	≥4	39.5	74.6	1.6	0.8	0.615
**B) IN-HOSPITAL MORTALITY**						
	qSOFA*					
	≥1	15.5 (14.9)	87.5 (89.5)	1.2 (1.4)	1.0 (1.0)	
	≥2	26.6 (24.4)	88.3 (88.9)	2.3 (2.2)	0.8 (0.9)	
	≥3	50.0 (54.5)	86.6 (87.5)	3.7 (4.4)	1.9 (0.5)	0.587 (0.592)
	Suspected sepsis*					
	yes	14 (18.6)	85.5 (86.9)	0.9 (1.4)	1 (0.9)	0.553 (0.527)
	**CURB-65**					
	≥1	15.9	92.1	2.0	0.9	
	≥2	19.2	90.9	2.1	0.9	
	≥3	30.2	89.0	2.8	0.8	
	≥4	35.7	86.4	2.6	0.8	
	≥5	40.0	85.9	2.8	0.7	0.650
	**SIRS**					
	≥1	18.8	86.5	1.4	0.9	
	≥2	15.7	97	5.2	0.9	
	≥3	14	84.6	0.9	1	
	≥4	13.4	84.9	0.9	1	0.497

* corresponding predictive values of the full set (n = 527) are shown in brackets

No significant differences were found between the AUC for in-hospital mortality or ICU admissions for qSOFA vs. SIRS (p = 0.054 / p = 0.143) or for qSOFA vs. clinically suspected sepsis (p = 0.186 / p = 0.262).

qSOFA had a significantly higher AUC than CURB-65 in predicting ICU admission (p = 0.013), but not in predicting in-hospital mortality (p = 0.156).

### Comparison of included and excluded patients

In the primary outcome analysis, 13.9% (n = 85) of the eligible patients had to be excluded because of missing data, which was mainly a missing documented respiratory rate (n = 77). There was no significant association between being excluded and any studied risk factor for pneumonia (p>0.1). Furthermore, no significant differences were found in regard to in-hospital mortality (p = 0.221) or ICU admission (p = 0.396). The length of hospitalisation was significantly (p = 0.030) increased in the included patients (median 8, IQR 5–14) compared to the excluded patients (median 7, IQR 4–11).

## Discussion

### Key findings

This study analysed the predictive performance of qSOFA in ED patients with pneumonia in regard to in-hospital-mortality, ICU admission and length of hospital stay. The predictive ability of qSOFA was presented and compared to other outcome scores in a subgroup analysis.

Our results show that qSOFA predicts in-hospital mortality, ICU admission, and length of stay in patients admitted with pneumonia. The sensitivity of qSOFA was low. qSOFA was superior to CURB-65 in predicting ICU admissions in ED patients with pneumonia. The evaluated scores were equally effective in respect to in-hospital mortality.

### qSOFA assessment

Several recently published trials investigated the predictive performance of qSOFA in patients in the general emergency department population with suspected infection [7, 16, 17, 27–30]; some of these studies also investigated patients with pneumonia [17, 28, 30]. Other authors have shown that qSOFA has significant predictive ability in respect to in-hospital mortality in patients with pneumonia [17, 28, 30]. However, the AUC values in published studies on this topic are highly disparate [17, 28, 30]. Although our study only showed a moderate AUC for in-hospital mortality in patients with pneumonia, others have found AUCs ranging from 0.7 to 0.81 for prediction of in-hospital mortality [17, 28, 30].

Two studies were restricted to community-acquired pneumonia in Germany and Spain and found an AUC for 30-day-mortality of 0.70 [28] and in-hospital-mortality of 0.69 [30]. The higher AUC might be explained by the exceptionally low mortality rates (4% and 6%) in these studies [28, 30]. The latter can be explained by the restriction to community-acquired pneumonia only, whereas in our study 9.7% of the patients suffered from nosocomial pneumonia, which is associated with a higher mortality rate [31].

A further study set in China even found an AUC of 0.81 for 28-day mortality [17]. Although the high AUC in the latter study is striking, the results are not comparable to Western studies, as the 28-day-mortality for hospitalised patients was extremely high (54%), with ICU admission of 85% [17].

Despite the good predictive ability of qSOFA in general ED patients [7, 16, 27], the prognostic accuracy of the qSOFA score was much smaller for in-hospital mortality among adults admitted to the ICU with suspected infection [29]. In the ICU setting, the SOFA score shows the best predictive ability [29]. Remarkably however, in ICU patients, the AUC (0.607) of a positive qSOFA in predicting in-hospital mortality [29] was very close to the findings in this study (0.608), whereas the AUC for predicting mortality was significantly higher in less ill patient populations [7, 16]. Taken together, these results may indicate that qSOFA is more useful in predicting mortality in populations with lower mortality rates.

As found by other authors, qSOFA has low sensitivity in respect to the outcomes in this study [17, 30, 32]. Nonetheless, the sensitivity is comparable to that of other scores, such as CURB-65 or SIRS.

qSOFA score was associated with ICU admission in this study. This is in accordance with other studies on general ED patients admitted with suspected infection [7, 16], and also with a study that investigated patients presenting with pneumonia [17].

qSOFA score is associated with the length of stay in hospital in this study. While it has been shown that qSOFA was associated with the length of hospitalisation in ICU patients with suspected infection [29], this is the first study to show an association with length of stay in ED patients.

### Comparison of qSOFA to other outcome prediction scores

To our knowledge, this is the first study that compared CURB-65 and qSOFA in regard to ICU admissions in ED patients diagnosed with pneumonia. One other study compared CRB and CRB-65 with qSOFA in respect to ICU admissions, without taking into account blood urea nitrogen [17]. While the authors of this study found that the number of ICU admissions was higher when q-SOFA was used for predicting ICU admission than with CRB-65(p<0.01), no significant difference (p>0.05) between these two scores was found in the AUC, which ranged from 0.57 to 0.68 (only range given). The difference might be explained by differences in the procedures for ICU admission procedures in other health systems and the relatively low hospitalisation rate of (53%) in the study by Chen et al. [17], which indicates that their study population might have been different from ours. In the setting of a university hospital in Europe, our result suggest, that qSOFA is superior to CURB-65 in predicting ICU admission.

qSOFA and CURB-65 score are equal in their predictive abilities for in-hospital mortality. However, the AUC for in-hospital mortality for CURB-65 was non-significantly higher than the AUC for qSOFA. Other studies also found non-significant differences, with higher AUC for CURB-65 [17, 28]. This should certainly be further investigated, as qSOFA has the major advantage that it requires no laboratory work-up to calculate the score. It could then be used for the bedside assessment of patients presenting with pneumonia within minutes of arrival. CURB-65 on the other hand needs a urea value, which takes roughly one hour of laboratory work-up, in addition to the time spent to draw the blood sample. This may waste essential time in managing critically ill patients with pneumonia. However, time is a crucial issue when treating patients with sepsis. qSOFA therefore might provide a more rapid and cheaper alternative to evaluating disease severity in patients presenting with pneumonia. The addition of an age component (one point for an age of 65 or more) to qSOFA for instance increased its predictive ability for 30-day mortality in patients with pneumonia [28].

Surprisingly, SIRS criteria were not associated with in-hospital mortality in this study. Two studies have compared SIRS criteria with qSOFA, one on patients with suspected infection outside the ICU (7) and another very recently published study on patients with pneumonia [30]. These showed that qSOFA was superior to SIRS criteria in the prediction of mortality. In our study, the AUC for in-hospital mortality was higher in qSOFA than with SIRS, but the difference was not significant. In a study that compared SIRS, qSOFA and SOFA in respect to in-hospital mortality in ICU patients, SOFA gave significantly better results, with no difference between SIRS and qSOFA [29]. Thus, qSOFA may be superior to SIRS criteria for predicting in-hospital mortality in ED patients with pneumonia. However, on the ICU, SOFA might be the optimal choice to investigate in-hospital mortality.

### Study limitations and strength

Our study has several limitations. Firstly, the study was retrospective design and used medical records. Thus, no standardised form for each patient was completed at the time of admission. It is therefore possible that risk factors or symptoms were not recorded by the treating physician, even though they were present in the patient. This also led to a significant percentage of excluded patients due to incomplete data sets for the calculation of qSOFA. However, in the main analysis, a comparison of in- vs. excluded patients did not show significant differences in regard to in-hospital mortality or risk factors for pneumonia, so that selection bias is unlikely. On the other hand, in the subgroup analysis of all patients with complete data to calculate SIRS criteria and CURB-65 –blood urea nitrogen and respiratory rate were often missing.

The case definition of pneumonia was based on the diagnosis documented in the medical report of the patient. While the diagnosis is usually based on radiography and the clinical presentation of the patient, a diagnosis bias cannot be excluded. This could be a source of a possible selection bias.

The results are restricted to patients primarily diagnosed with pneumonia in the ED. Furthermore, treatment restriction was not taken into account, which might have biased the association of ICU admission and the different scores.

## Conclusions

qSOFA predicts in-hospital mortality, length of hospitalisation, and ICU admission in ED patients presenting with pneumonia. qSOFA is superior to CURB-65 in respect to ICU admissions. In respect to in-hospital mortality, the evaluated scores were equivalent. qSOFA has the advantage of being a pure bedside test. As it requires no laboratory testing, it may be more practical than the CURB-65 score or SIRS criteria for routine clinical work.

## Supporting information

S1 Dataset The dataset of this study (n = 527). (XLSX)
